# Health-Seeking Influence Reflected by Online Health-Related Messages Received on Social Media: Cross-Sectional Survey

**DOI:** 10.2196/jmir.5989

**Published:** 2017-11-16

**Authors:** Rahila Iftikhar, Bahaa Abaalkhail

**Affiliations:** ^1^ Department of Family and Community Medicine King Abdulaziz University Jeddah Saudi Arabia

**Keywords:** online health information-seeking behaviors, Facebook, social media, Twitter, WhatsApp

## Abstract

**Background:**

Major social networking platforms, such as Facebook, WhatsApp, and Twitter, have become popular means through which people share health-related information, irrespective of whether messages disseminated through these channels are authentic.

**Objective:**

This study aims to describe the demographic characteristics of patients that may demonstrate their attitudes toward medical information shared on social media networks. Second, we address how information found through social media affects the way people deal with their health. Third, we examine whether patients initiate or alter/discontinue their medications based on information derived from social media.

**Methods:**

We conducted a cross-sectional survey between April and June 2015 on patients attending outpatient clinics at King Abdulaziz University, Jeddah, Saudi Arabia. Patients who used social media (Facebook, WhatsApp, and Twitter) were included. We designed a questionnaire with closed-ended and multiple-choice questions to assess the type of social media platforms patients used and whether information received on these platforms influenced their health care decisions. We used chi-square test to establish the relationship between categorical variables.

**Results:**

Of the 442 patients who filled in the questionnaires, 401 used Facebook, WhatsApp, or Twitter. The majority of respondents (89.8%, 397/442) used WhatsApp, followed by Facebook (58.6%, 259/442) and Twitter (42.3%, 187/442). In most cases, respondents received health-related messages from WhatsApp and approximately 42.6% (171/401) reported ever stopping treatment as advised on a social media platform. A significantly higher proportion of patients without heart disease (*P*=.001) and obese persons (*P*=.01) checked the authenticity of information received on social media. Social media messages influenced decision making among patients without heart disease (*P*=.04). Respondents without heart disease (*P*=.001) and obese persons (*P*=.01) were more likely to discuss health-related information received on social media channels with a health care professional. A significant proportion of WhatsApp users reported that health-related information received on this platform influenced decisions regarding their family’s health care (*P*=.001). Respondents’ decisions regarding family health care were more likely to be influenced when they used two or all three types of platforms (*P*=.003).

**Conclusions:**

Health education in the digital era needs to be accurate, evidence-based, and regulated. As technologies continue to evolve, we must be equipped to face the challenges it brings with it.

## Introduction

Twitter, Facebook, and WhatsApp have become mainstream online tools that permit individuals to connect and share information. Furthermore, they permit individuals to share uncontrolled, unsupervised, and unfiltered content, irrespective of time and place [1]. Consequently, the Internet contains a lot of self-created content [2]. Many people are increasingly using social networking sites for health-related purposes. Research has demonstrated that an increasing number of patients are using social networking sites to share their experiences with health care personnel or institutions [3]. Patients also share their experiences with family members and friends via platforms such as Facebook, WhatsApp, and Twitter [4-6]. There is evidence that if social networking is used properly, then it can help patients [7]. For example, a study that promoted breastfeeding among Saudi women showed increased adherence to breastfeeding through a Twitter campaign [8]. Another study that investigated the effect of Twitter on women’s health education demonstrated that women in Saudi Arabia were interested in discussing gynecological complains and breastfeeding-related issues on Twitter [9]. The investigators found that this strategy helped in creating awareness. Moreover, a recent study showed that Twitter was a powerful platform for health promotion strategies [10]. Influential people who have a huge number of followers can constitute an integral part of any health campaign or help in disseminating knowledge.

Concerns about the increasing use of social media to share health experiences and information arise as the use of these sites might affect choices that patients make regarding their health [11]. Furthermore, it might affect the way patients interact with health care professionals. According to one review, information obtained from social networking sites correlated with many measures of quality of care, including performance measures such as mortality and readmission rates [12]. Nevertheless, definitive conclusions cannot be drawn from correlation tests and several questions remain unanswered regarding the impact of patients’ use of social media.

There are few data available regarding the impact of social networking sites on the online health information-seeking behaviors of people in Saudi Arabia [13]. This report will attempt to determine whether advice obtained from social media platforms, such as Facebook and WhatsApp, affect choices that people make about their health care. This study aims to describe the demographic characteristics of patients that may demonstrate their attitudes toward medical information shared on social media networks. Second, we address how information found through social media affects the way people deal with their health. Third, we examine whether patients initiate or alter/discontinue their medications based on information derived from social media.

## Methods

### Participants and Setting

A cross-sectional survey was conducted between April and June 2015 on patients attending King Abdulaziz University Hospital, Jeddah, Saudi Arabia. We included patients who used Facebook, WhatsApp, and Twitter, which are among the most frequently used social media platforms in Saudi Arabia [14]. Informed consent was obtained from all participants prior to recruitment. The Research Ethics Committee at King Abdulaziz University Hospital approved the study.

The initial pool included 442 participants of which 401 reported using Facebook, WhatsApp, or Twitter. The demographics reported reflect the 442 patients who agreed to participate in this survey; however, further analysis was performed only for the 401 participants who used any of the social media platforms under consideration.

### Survey Instrument

We modified a previously validated questionnaire [15] to specifically target social media users and how medical information found through social media networks impacts the way they deal with their health. Furthermore, we selected highly prevalent public health issues in Saudi Arabia, such as diabetes mellitus, heart disease, hypertension, and asthma.

The questionnaire was developed with closed-ended and multiple-choice questions that were designed to be nonintrusive and simple to understand. The questionnaire was administered by medical students, who interviewed the participants. Prior to the interview, the students were trained to collect data. Participants were asked to identify their age, gender, nationality, marital status, educational level, and monthly income. They were also asked the types of social media platforms they used and whether information received on these platforms influenced their health care decisions. Returned questionnaires were reviewed and those that were filled in by respondents who did not use any of the three social media platforms (Facebook, WhatsApp, or Twitter) were excluded from analysis.

### Statistical Analysis

The data were analyzed using IBM SPSS version 22. Descriptive statistics were computed for all variables. The findings are expressed as counts and percentages for categorical and nominal variables, whereas continuous variables are presented as means and standard deviations. To establish the relationship between categorical variables, chi-square test was used. This test was conducted with the assumption of normal distribution. Lastly, a conventional *P* value <.05 was adopted to reject the null hypothesis.

## Results

### Demographic Characteristics

A total of 442 participants with a mean age of 35.4 (11.5) years filled in the questionnaires. Females comprised the majority of the sample (256/442, 71.9%). Approximately 46.0% (193/420) of the respondents had completed at least university education (Table 1). Regarding income, 97 respondents reported incomes greater than 10,000 Saudi riyals (US $2666). Approximately 74.9% (328/438) of the respondents were married and 59.8% (259/433) were Saudis (Table 1).

### Type of Social Media Platforms Used

Approximately 90% (397/442) of the respondents used WhatsApp; Twitter was the least used among all three social media platforms. Approximately 32% (142/442) of respondents used all three types of social media platform (Table 2). Respondents received health-related messages more frequently on WhatsApp than Twitter or Facebook. Close to one-third of the respondents reported using all three social media platforms and respondents reported receiving health-related messages more frequently on WhatsApp than on Twitter or Facebook. Despite the number of respondents who reported receiving medical information through social media, less than one-fifth admitted that information shared across these platforms always influenced their health decisions. Further, one-quarter of the respondents admitted to never discussing health-related information with their physicians.

**Table 1:** Demographic characteristics of the sample (N=442).

Variables		n (%)^a^
**Gender**		
	Male	123 (27.8)
	Female	319 (72.2)
**Age group (years)**		
	≤29	138 (31.2)
	30-39	123 (27.8)
	40-49	104 (23.5)
	50-59	49 (11.1)
	≥60	28 (6.3)
**Marital status**		
	Married	328 (74.9)
	Single	110 (25.1)
**Nationality**		
	Saudi	259 (59.8)
	Non-Saudi	174 (40.2)
**Educational attainment**		
	Primary	56 (13.3)
	Secondary	158 (37.6)
	Graduation/Postgraduation	193 (46.0)
	None	13 (3.1)
**Occupation**		
	Housewife	190 (44.8)
	Office job	41 (9.7)
	Business	19 (4.5)
	Doctor	12 (2.8)
	Engineer	8 (1.9)
	Unemployed	27 (6.4)
	Others	127 (30)
**Monthly income in Saudi riyals (US$)**		
	<2000 (533)	46 (11.4)
	2000-5000 (533-1333)	135 (33.3)
	5000-10,000 (1333-2666)	127 (31.4)
	10,000-20,000 (2666-5333)	76 (18.8)
	>20,000 (5333)	21 (5.2)

^a^Some cases have missing values.

**Table 2:** Usage and type of social media used among the respondents (N=442).

Variables		n (%)^a^
**Type of social media platform used**		
	WhatsApp	397 (89.8)
	Facebook	259 (58.6)
	Twitter	187 (42.3)
**Number of social media platforms used**		
	Never use	41 (9.3)
	Any of one	101 (22.9)
	Any of two	158 (35.7)
	All types	142 (32.1)
**Do you receive health-related messages on WhatsApp?**		
	Yes	311 (78.3)
	No	86 (21.7)
**Do you receive health-related messages on Facebook?**		
	Yes	121 (46.7)
	No	138 (53.3)
**Do you receive health-related messages on Twitter?**		
	Yes	125 (66.8)
	No	54 (28.9)
	Not reported	8 (4.3)
**Do messages on social media platforms ever influence your decisions regarding you or your family’s health care?**		
	Always	65 (16.2)
	Sometimes	185 (46.1)
	Never	116 (28.9)
	Not reported	35 (8.7)
**Do you discuss the authenticity or usefulness of health-related information received on social media platforms with a doctor or other health care professionals?**		
	Always	128 (31.9)
	Sometimes	128 (31.9)
	Never	104 (25.9)
	Not reported	41 (10.2)

^a^Some cases have missing values, and some respondents used more than one social media platform.

### Practices of the Participants Regarding Health-Related Information Received on Social Media Platforms

Among the 401 participants who used Facebook, WhatsApp, or Twitter, less than half admitted starting a treatment as advised on social media without asking their physician (Table 3). In most cases, respondents received health-related messages from WhatsApp and 42.6% (171/401) reported ever stopping treatment as advised on a social media platform. Approximately half (86/171) of respondents were mostly influenced by WhatsApp. Close to one-fifth of the respondents never verified the credibility of the health information received on social media platforms and about one-quarter shared the information without verifying whether it was accurate. Google was cited as the main site where people performed searches to verify the accuracy of health-related information received on social media platforms.

### Discussion of Information Received on Social Media With Health Care Professionals Stratified by Health Status

A significantly higher proportion of patients without heart disease (*P*=.001) and obese persons (*P*=.01) checked the authenticity of information received through social medial channels (Table 4). There were no differences between persons with diabetes, hypertension, asthma, dyslipidemia, chronic disease, and those without any of these conditions.

**Table 3:** Practices of social media users regarding information received on social media platforms (N=401).

Variables		n (%)^a^	
**Have you ever started any medications/treatment as advised/advertised on social media without asking your physician?**			
	Yes		183 (46.6)
	No		210 (53.4)
**If yes, which social media platform influenced you most?**			
	WhatsApp		93 (50.8)
	Facebook		24 (13.1)
	Twitter		32 (17.5)
	Not reported		34 (18.6)
**Have you ever stopped any medications/treatment as advised/advertised on social media without asking your physician?**			
	Yes		171 (42.6)
	No		221 (55.1)
	Not reported		9 (2.2)
**If yes, which social media platform influenced you most?**			
	WhatsApp		86 (50.3)
	Facebook		17 (9.9)
	Twitter		29 (17.0)
	Not reported		39 (22.8)
**Do you verify the credibility of the health information on social media?**			
	Always		146 (40.7)
	Sometimes		149 (41.5)
	Never		64 (17.8)
**If yes, which sources do you mostly use for verification?**			
	Google		259 (90.9)
	Others (PubMed/Ministry of Health website, etc)		26 (9.1)
**Do you verify the credibility of health-related information before sharing it with other people?**			
	Always		150 (42.3)
	Sometimes		116 (32.7)
	Never		89 (25.1)

^a^Some cases have missing values.

**Table 4:** Discussion of information received on social media with health care professionals stratified by health status.

Variables		Discuss the authenticity of information read on social media platforms with health care professionals, n (%)		*P*
		Yes (n=256)	No (n=104)	
**Do you have diabetes?**				.24
	Yes	38 (64.4)	21 (35.6)	
	No	199 (72.1)	77 (27.9)	
**Do you have heart disease?**				.001
	Yes	5 (33.3)	10 (66.7)	
	No	231 (72.9)	86 (27.1)	
**Do you have hypertension?**				.46
	Yes	35 (76.1)	11 (23.9)	
	No	203 (70.7)	84 (29.3)	
**Do you have asthma?**				.66
	Yes	23 (67.6)	11 (32.4)	
	No	213 (71.2)	86 (28.8)	
**Are you obese?**				.01
	Yes	42 (87.5)	6 (12.5)	
	No	198 (70.0)	85 (30.0)	
**Do you have dyslipidemia (increased cholesterol)?**				.37
	Yes	29 (65.9)	15 (34.1)	
	No	211 (72.5)	80 (27.5)	
**Do you have any other disease?**				.74
	Yes	72 (74.2)	25 (25.8)	
	No	168 (72.4)	64 (27.6)	
**Do you have any chronic disease?**				.44
	Yes	108 (68.8)	49 (31.2)	
	No	140 (72.5)	53 (27.5)	

### Association Between Demographic Characteristics and Participants’ Attitudes Toward Health-Related Information Shared on Social Media Networks

There was no association between demographics and whether messages ever influenced family health care (Table 5). Married respondents did not differ significantly from single respondents in their attitudes toward medical information shared on social media (*P*=.99). Although participants who had completed secondary education were more likely than primary school leavers and university graduates to be influenced by health messages posted on social media, this difference was not significant (*P*=.52). Similarly, no association was found between respondents’ attitudes toward medical information posted on social media and sociodemographic variables, such as occupation (*P*=.95), gender (*P*=.81), nationality (*P*=.53), monthly incomes (*P*=.95), and age (*P*=.31).

### Association Between Participants’ Health Status and Their Attitudes to Medical Information Shared on Social Media Platforms

Decision making by patients without heart disease was influenced by social media messages (*P*=.04), whereas patients with other chronic diseases were not significantly influenced (*P*=.50). Respondents without heart disease (*P*=.001) and obese persons (*P*=.01) were more likely to discuss health-related information received on social media channels with health care professional. Patients with diabetes, hypertension, asthma, dyslipidemia, or those with any other chronic disease did not differ regarding their likelihood to discuss online health information with health care personnel. Furthermore, persons without diabetes (*P*=.04) or without heart disease (*P*=.001) tended to verify the credibility of information posted on social media channels (Table 6).

**Table 5:** Association between demographic characteristics and participants’ attitudes toward health-related information shared on social media networks^a^.

Variables		Messages ever influence decisions regarding family’s health care, n (%)				Verify the credibility of the health information on social media from other authentic sources, n (%)		
		Yes (n=250)	No (n=116)	*P*	Yes (n=295)		No (n=64)	*P*
**Gender**				.08				.58
	Male	72 (69.2)	32 (30.8)		82 (80.4)		20 (19.6)	
	Female	178 (67.9)	84 (32.1)		213 (82.9)		44 (17.1)	
**Age group (years)**				.31				.73
	≤29	76 (62.3)	46 (37.7)		101 (83.5)		20 (16.5)	
	30-39	85 (75.2)	28 (24.8)		88 (80.0)		22 (20.0)	
	40-49	59 (67.8)	28 (32.2)		73 (84.9)		13 (15.1)	
	50-59	19 (65.5)	10 (34.5)		23 (82.1)		5 (17.9)	
	≥60	11 (73.3)	4 (26.7)		10 (71.4)		4 (28.6)	
**Marital status**				.86				.72
	Married	181 (68.3)	84 (31.7)		212 (81.9)		47 (18.1)	
	Single	67 (68.4)	31 (31.6)		81 (83.5)		16 (16.5)	
**Nationality**				.53				.68
	Saudi	146 (66.4)	74 (33.6)		176 (81.1)		41 (18.9)	
	Non-Saudi	96 (69.6)	42 (30.4)		111 (82.8)		23 (17.2)	
**Education**				.52				.02
	Primary	21 (63.6)	12 (36.4)		20 (64.5)		11 (35.5)	
	Secondary	94 (70.1)	40 (29.9)		103 (78.6)		28 (21.4)	
	Graduation/Postgraduation	122 (68.9)	55 (31.1)		154 (87.0)		23 (13.0)	
	None	1 (33.3)	2 (66.7)		1 (100.0)		0 (0.0)	
**Employment**				.95				.15
	Unemployed	120 (69.8)	52 (30.2)		130 (78.8)		35 (21.2)	
	Employed	124 (70.1)	53 (29.9)		151 (84.8)		27 (15.2)	
**Monthly income in Saudi riyals (US $)**				.95				.36
	<5000 (1333)	94 (69.1)	42 (30.9)		109 (80.7)		26 (19.3)	
	5000-10,000 (1333-2666)	76 (67.3)	37 (32.7)		93 (86.1)		15 (13.9)	
	>10,000 (2666)	61 (68.5)	28 (31.5)		70 (78.7)		19 (21.3)	

^a^The total is <401 in some cases due to missing responses.

**Table 6:** Association between participants’ health status and their attitudes toward information found through social media^a^.

Variables			Messages on social media platforms influence decisions regarding family’s health care, n (%)						Verify the credibility of the health information on social media from other authentic sources, n (%)				
			Yes (n=250)		No (n=116)		*P*		Yes (n=295)		No (n=64)		*P*
**Do you have diabetes?**							.66						.04
	Yes	38 (65.5)		20 (34.5)				40 (71.4)		16 (28.6)			
	No	193 (64.8)		89 (31.6)				230 (83.0)		47 (17.0)			
**Do you have heart disease?**							.04						.001
	Yes	6 (42.9)		8 (57.1)				8 (50.0)		8 (50.0)			
	No	222 (68.9)		101 (31.3)				259 (82.5)		55 (17.5)			
**Do you have hypertension?**							.78						.06
	Yes	32 (69.6)		14 (30.4)				31 (70.5)		13 (29.5)			
	No	197 (67.5)		95 (32.5)				237 (82.6)		50 (17.4)			
**Do you have asthma?**							.43						.80
	Yes	25 (73.5)		9 (26.5)				29 (82.9)		6 (17.1)			
	No	203 (66.8)		101 (33.2)				240 (81.1)		56 (18.9)			
**Are you obese?**							.30						.68
	Yes	34 (73.9)		12 (26.1)				40 (83.3)		8 (16.7)			
	No	192 (66.2)		98 (33.8)				227 (80.8)		54 (19.2)			
**Do you have dyslipidemia?**							.71						.33
	Yes	29 (70.7)		12 (29.3)				32 (76.2)		10 (23.8)			
	No	203 (67.9)		96 (32.1)				240 (82.5)		51 (17.5)			
**Do you have any other disease?**							.06						.27
	Yes	61 (62.2)		37 (37.8)				79 (79.8)		20 (20.2)			
	No	172 (72.6)		65 (27.4)				195 (85.8)		35 (15.2)			
**Do you have any chronic disease?**							.50						.23
	Yes	150 (69.8)		65 (30.2)				121 (79.1)		32 (20.9)			
	No	99 (66.4)		50 (33.6)				164 (84.1)		31 (15.9)			

^a^The total is <401 due to missing responses.

**Table 7:** Association between social media type and participants’ attitudes toward medical information shared through social media^a^.

Variables			Messages on social media platforms influence decisions regarding family’s health care, n (%)						Discuss authenticity of message with health care professionals, n (%)					Verify the credibility of the health information on social media from other authentic sources, n (%)					
			Yes (n=250)		No (n=116)		*P*		Yes (n=256)		No (n=104)		*P*		Yes (n=295)		No (n=64)		*P*
**Do you use WhatsApp?**							.001						.19						.24
	Yes	221 (71.5)		88 (28.5)				221 (72.5)		84 (27.5)				250 (83.1)		51 (16.9)			
	No	26 (48.1)		28 (51.9)				33 (63.5)		19 (36.5)				42 (76.4)		13 (23.6)			
**Do you use Facebook?**							.53						.05						.02
	Yes	87 (72.5)		33 (27.5)				91 (75.8)		29 (24.2)				108 (90)		12 (10.0)			
	No	84 (68.9)		38 (31.1)				78 (64.5)		43 (35.5)				94 (79)		25 (21.0)			
**Do you use Twitter?**							.86						.05						.03
	Yes	88 (71.0)		36 (29.0)				92 (74.8)		31 (25.2)				109 (89.3)		13 (10.7)			
	No	32 (69.6)		14 (30.4)				28 (59.6)		19 (40.4)				36 (76.6)		11 (23.4)			
**How many of these social media platforms (Facebook, Twitter, or WhatsApp) do you use?**						.003						.12						.10	
	Only 1	54 (53.5)		47 (46.5)				64 (78)		18 (22.0)				64 (76.2)		20 (23.8)			
	2 or all 3	196 (65.3)		104 (34.7)				192 (69.1)		86 (30.9)				231 (84)		44 (16.0)			

^a^The total is <401 due to missing responses.

### Impact of Social Media Platform Used on Participants’ Attitudes Toward Health-Related Information Shared on These Platforms

A significant proportion of WhatsApp users reported that health-related information disseminated on this platform influenced decisions regarding their family’s health care (*P*=.001; Table 7). Similarly, respondents’ decisions regarding family health care were more likely to be influenced when they used two or all three types of platforms (*P*=.003). Respondents’ decisions regarding family health care did not differ significantly between those who used Facebook or Twitter and those who did not use these platforms.

## Discussion

In this study, we explored the impact of health-related information sharing, the influence of social media on peoples’ online health information-seeking behavior, and their diligence in following prescriptions, as well as self-medication among social media users. This study shows that most people (89.8%, 397/442) used WhatsApp and 78.3% (311/397) of social media users received health information through these channels. Less than one-fifth of social media users admitted that health-related messages received on these platforms always influenced their decisions regarding family members’ health care. Furthermore, a large proportion of patients (46.6%, 186/393) admitted starting medications as advertised on social media platforms without consulting a physician. Similarly, 42.6% (171/401) of patients stopped taking their medication after reading messages received on a social media platform.

It is unquestionable that health care and allied health professionals can use the power of social media to spread information, including recruiting patients for clinical studies and surveying patients to get their opinions on a new treatment or device; however, potential risks may ensue from the use of social media when there are no stringent regulations to share and receive health care information on these platforms. Several investigators have expressed concerns about the potential of social media to negatively impact patients and their treatment [16,17]. In this study, for example, approximately half of respondents who either started or stopped medication were influenced by WhatsApp, reflecting the importance of the way this platform influences how people deal with their health. Less than half the respondents always verified the credibility of information and 90.9% (259/285) performed a Google search to verify the authenticity of messages received through social media channels. Interestingly, 25.1% (89/355) of respondents never discussed health-related messages with their physicians. This might be due to the fact they did not have a regular physician or they did not find it relevant to discuss this with a health care professional. Furthermore, women were more likely to discuss health-related information with their physician for authenticity as compared to men. Another study demonstrated that young male patients sought medical help less frequently and tended to avoid medical consultations [18].

We found that patients who had attained postgraduate college degrees were more likely to verify the credibility of information received via social media channels. According to a previous systematic review [19], educational status appeared to affect the way people evaluated online health information, with individuals with a lower level of education demonstrating worse capacities to evaluate the authenticity of health information shared on social media and lower trust in online health information compared to their more educated peers. Regarding perceived quality of online health information or people’s use of evaluation criteria, the limited number of studies and the diversity of samples and measures do not allow us to draw conclusions about the impact of educational level or other skills-based proxies of health literacy leaving two of the main research questions of this study mainly unanswered. Similarly, we found that patients without diabetes or heart disease were more likely to verify the credibility of medical information shared on social networks. This suggests that patients who have diabetes and heart disease are less likely to verify the authenticity of health information received on social media. This is a concerning factor because patients with chronic diseases should seek medical advice and have regular follow-ups with their doctors and, consequently, should have better education regarding their disease.

The Internet and social media, in particular, provide a business platform to pharmaceutical companies and, according to a recent survey, 40% of top pharmaceutical companies use direct-to-consumer advertising on social media platforms [20]. In the clinical scenario, a physician has to balance the risks against the benefits of prescribing a particular diagnostic test or therapy. Hence, their clinical decisions are based on the patient’s understanding, informed consent after explanation of potential risks, preferences, and available resources. On the contrary, social media and other online platforms, which are typically unregulated, may pose a potential threat to patient safety by encouraging the illegal online nonmedical use of prescription drugs [21]. In our setting, for example, we have noticed that patients tend to self-medicate and use complementary medicines. They often get health-related messages on mammography or prostate cancer screening and request to have these investigations without actually understanding the risk or benefit for such diagnostic tests or particular treatment.

Several studies [21-26] have stated that social media has a positive effect on health care, including mental health and physical fitness programs. In fact, it has been suggested that the use of social networking sites to share credible health information, can help physicians fulfill the professional obligation to transmit pertinent information to patients, colleagues, and the public and help members of the public place the findings of health-related current events in proper context. Some physicians affirm that physicians have an ethical obligation to lend their voices to public discourse on health care topics online [27]. Furthermore, it is believed that physicians who use their presence on social media to broadcast their professional commitments and values help fight the unscientific but amplified voices of the media and advertisements, which may disseminate spurious and sometimes dangerously incorrect statements regarding health [28].

We believe that there is an urgent need for mass awareness campaigns to educate people that medical information received on social media channels must be critically reviewed. People should be encouraged to consult their physicians prior to making any self-imposed changes to their prescriptions. Misinformation creates confusion and jeopardizes clinical care. Only 50% of television health shows give evidence-based advice [28], although hosts of television programs perform some degree of research before broadcasting. Similarly, a content analysis of information on urology disseminated on Facebook revealed that only 13% of the posts contained relevant information, whereas 40% were advertisements of commercial products [29]. In the same line, another study that assessed how health conditions were represented on Facebook pages revealed that 32.2% of the information was commercial, whereas 20% were about health awareness [30]. Therefore, it is important that social media users check the authenticity and relevance of all health-related information received on Facebook, Twitter, or WhatsApp. Moreover, there should be cyber surveillance as part of social accountability for spreading potentially incorrect health information. This can be possible by having health professionals edit social network pages to suit patients’ needs.

In our context, this study is the first to assess the impact of social media on the way people deal with their health and how messages received on social media platforms influence self-medication practices. However, our findings are limited because this study was conducted at a tertiary care hospital, which may not represent the community setting. Moreover, because we used a cross-sectional convenience sample, we could not establish how social networking affects patients’ health decisions.

Our findings indicate that social media is an important tool for health information. In addition, it influences people’s behaviors and self-medication practices. This suggests that clinicians need to assess patients’ medication histories during every visit. Because compliance to treatment is always an issue for patients with chronic diseases, social media adds another dimension to it. It may provide unauthenticated, misleading information and grounds for unjustified use of medications. Furthermore, the interpretation of messages on social media can be difficult, confusing, and may not be fully comprehended.

Future research should focus on specific diseases such as diabetes mellitus and hypertension, and on the patient’s reasons for self-medication. Emphasis should also be placed on the types of medications that patients initiate and those that they stop as well as the consequences associated with such practices. Studies should also explore platforms that patients trust most and how they prefer health information to be communicated to them. Moreover, the reasons underlying people’s reluctance to discuss health information and self-medication practices with a physician should be explored in qualitative studies.

Patients should be educated to review all health information skeptically. Policymakers and doctors should endeavor to formulate authenticated local languages, for example, here Arabic health literacy websites where patients can check the credibility of any health-related information received on social media platforms. Health care administrators should also look ahead to plan/forecast future medical care regarding how much and how far doctors wish to be involved in online patient care (digital clinics) [31] and how this will be regularized. Many ethical questions need to be answered before we communicate treatment on Twitter or Facebook.

In conclusion, health education in the digital era needs to be accurate, evidence-based, and regulated. As technology continues to evolve, we must be equipped to face the challenges it brings with it. The two main challenges in this regard include legislation and patient confidentiality. Social media cannot replace proper consultation, listening to nonverbal cues, touch, physical examination, exploring patients’ ideas, expectations, and individualized care. Therefore, decisions regarding major clinical care should be encouraged in the professional setting.
